# Myopericytoma in the Broad Ligament: A Rare Entity at a Rare Site

**DOI:** 10.7759/cureus.80603

**Published:** 2025-03-15

**Authors:** Paulina Costa, Angela Encarnação Sousa Silva, Maria Manuel Torrão, Rita Trovão Sousa, Ana Lanzinha

**Affiliations:** 1 Obstetrics and Gynecology, Unidade Local de Saúde do Médio Ave, Vila Nova de Famalicão, PRT

**Keywords:** adnexal mass, broad ligament, immunohistochemistry, myopericytoma, soft-tissue tumour

## Abstract

Myopericytoma (MPC) is a rare mesenchymal soft-tissue tumor that typically presents as a benign lesion. It is most commonly found on the skin of extremities, with infrequent involvement of deeper structures. Its rarity in visceral locations makes preoperative diagnosis challenging, and its hypervascular nature can raise suspicion of malignancy. We report the case of a perimenopausal woman referred to gynecology for evaluation of an incidental adnexal mass detected during a routine transvaginal ultrasound. Further assessment revealed a highly vascularized broad ligament mass of uncertain origin. Laparoscopic excision was performed, and histological examination identified a mesenchymal tumor without malignant features. Immunohistochemical analysis was crucial and confirmed the diagnosis of a MPC. This case highlights the diagnostic challenge, management, and prognosis of this uncommon entity.

## Introduction

Myopericytoma (MPC) is a rare mesenchymal soft-tissue tumor originating from the perivascular myoid cells [1]. It may occur at any age, although most cases are seen in middle-aged adults [2]. Most cases present as a painless, slow-growing mass [3]. It is usually a benign mass arising in the subcutaneous soft tissues of extremities, followed by trunk, head, and neck regions. Visceral organs and the central nervous system are rarely involved. Involvement of the urinary tract, including the kidney, bladder, ureter, lung, heart, and liver, has been described in case reports [4,5]. To our knowledge, there is only one paper describing three cases of MPC affecting the female genital tract, specifically in the uterus and ovary [4]. Although benign, it is often difficult to differentiate from malignant soft tissue neoplasms, with imaging studies typically revealing a well-defined hypervascularized lesion [2]. Histopathology is crucial for categorizing the lesion, revealing a well-circumscribed soft tissue neoplasm with prominent vascular structures composed of multilayered spindle cells in a concentric growth pattern around numerous blood vessels, without nuclear atypia or necrosis. Immunohistochemistry further strengthens the diagnosis, staining positive for smooth muscle actin (SMA), h-caldesmon, and desmin; CD34, S100, and keratin are usually negative [2,3]. Recurrence is rare but has been documented [4]. To the best of our knowledge, we present the first report of an MPC in the broad ligament.

This case report was previously presented as an e-poster at the European Society for Gynaecological Endoscopy 31st Annual Congress on October 2, 2022.

## Case presentation

A 54-year-old woman, in perimenopause, was referred to our gynecology department due to the incidental finding of an adnexal mass during a routine transvaginal ultrasound. She had no significant personal medical history. Regarding previous surgeries, she had undergone only Morton’s neuroma excision and tubal ligation. Her obstetric history included two pregnancies, both resulting in vaginal deliveries. The cervical cancer screening was up-to-date and normal.

**Figure 1:**
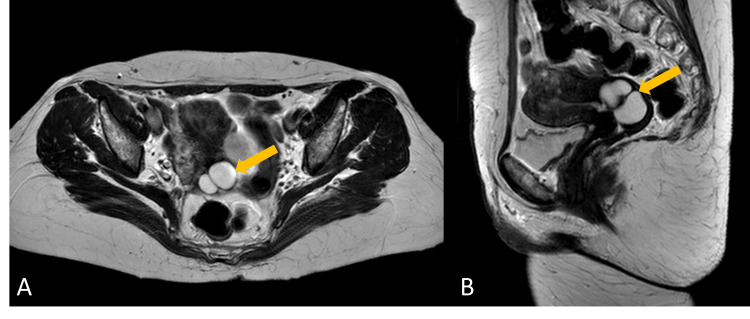
Post-contrast T2-weighted pelvic magnetic resonance imaging (lesion marked by yellow arrow) Image A shows a transverse view of the pelvis demonstrating an enhancing and well-defined lesion arising near the left side of the uterine isthmus and cervix. Image B shows a longitudinal view, illustrating the enhancing lesion in relation to the posterior-left side of the uterine isthmus and cervix.

The patient was asymptomatic, and the gynecological physical examination yielded no significant findings. The ultrasound identified an adnexal atypical mass that seemed to originate from the fallopian tube, measuring 58 x 19 mm in size. To better characterize the lesion, magnetic resonance imaging (MRI) was performed, revealing a highly vascularized left broad ligament mass of uncertain origin that measured 51 x 31 x 56 mm, apparently infiltrating the posterior-left uterine wall at the level of the isthmus and cervix (Figure 1).

**Figure 2:**
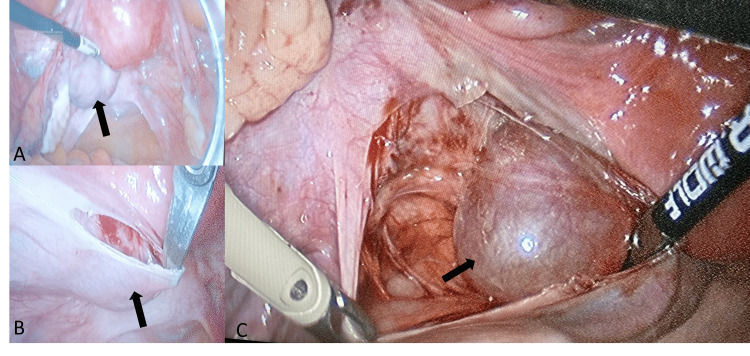
Laparoscopic view (lesion marked by black arrow) Image A shows a panoramic view of the pelvis revealing a well-defined mass in the posterior part of the left broad ligament, arising near the left side of the uterine isthmus and cervix, measuring approximately 4-5 cm in size. Image B shows the dissection of the mass, beginning with the opening of the posterior leaf of the broad ligament. Image C demonstrates the exposure of the lesion, revealing a smooth surface with no signs of invasion or necrosis macroscopically.

The preoperative study was normal and the serological neoplastic markers were negative. An exploratory laparoscopy confirmed a broad ligament mass located posteriorly and adjacent to the left side of the uterine isthmus and cervix, with a 4-5 cm diameter and elastic consistency (Figure 2).

**Figure 3:**
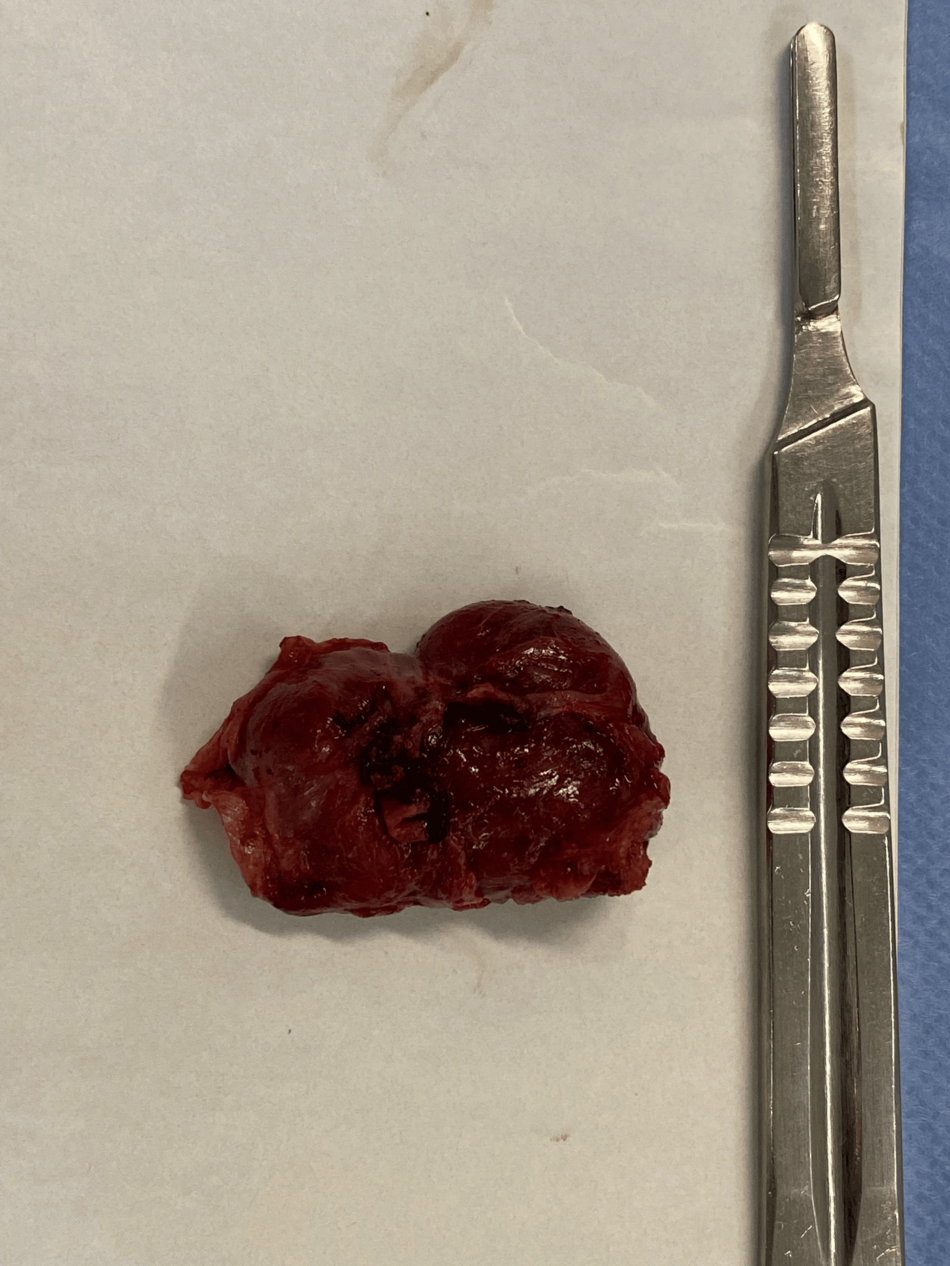
Macroscopic view after surgical excision Macroscopic view of the mass after excision showing a brown nodular mass with a smooth outer surface and elastic consistency.

We performed a laparoscopic excision of the lesion, without leaking, and the extemporaneous examination characterized the mass as a mesenchymal tumor, without malignant criteria (Figure 3).

**Figure 4:**
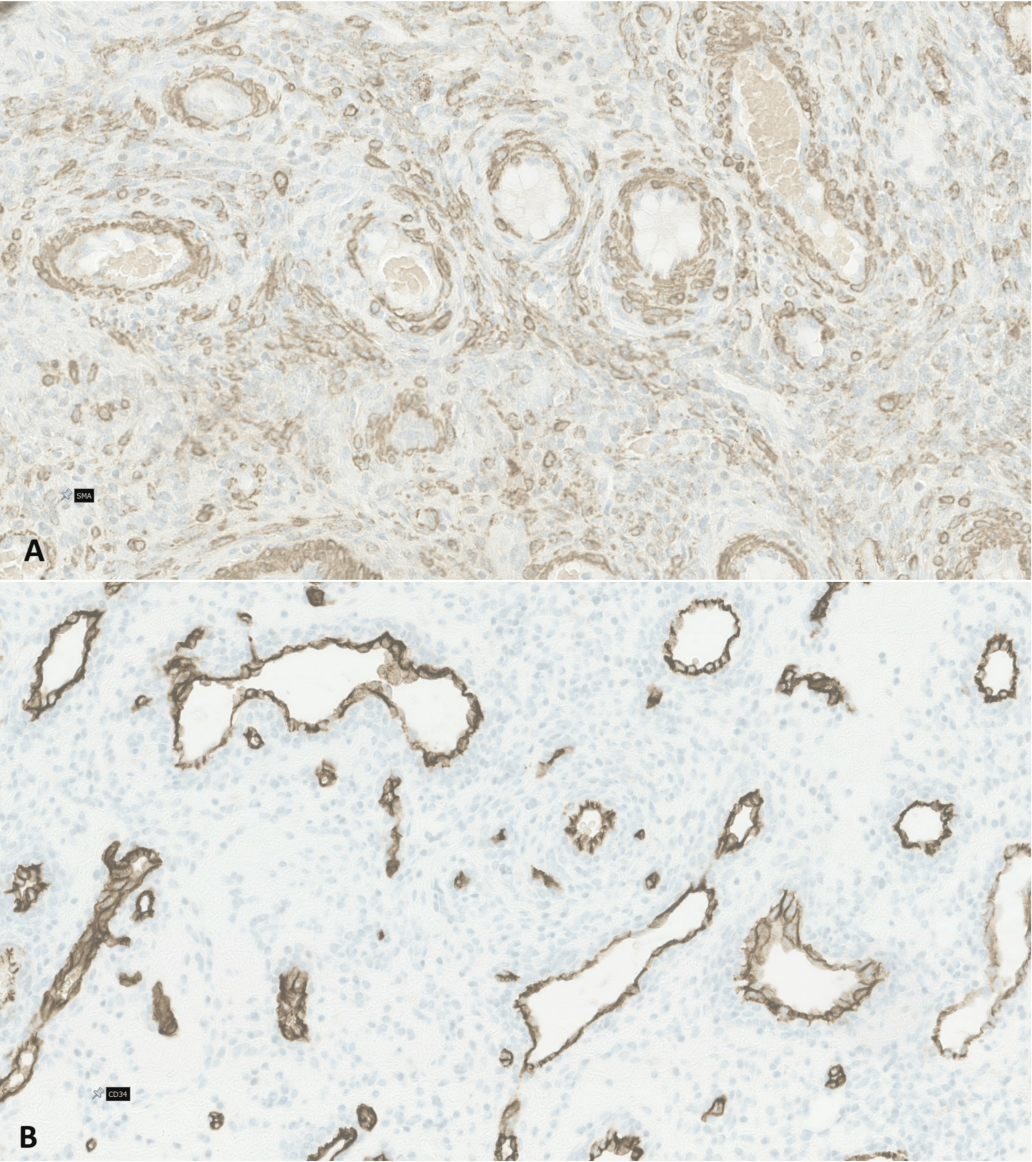
Images of the histological slides and immunohistochemical staining Both images demonstrate that the tumor exhibited no cellular atypia, pleomorphism, or necrosis. Image A shows smooth muscle actin positivity in the perivascular cells. Image B reveals CD34-positive staining in the endothelial cells of the blood vessels, but not in the tumor cells.

Surgery was undergone without complications, and the postoperative course was uneventful, with the patient being discharged the day after surgery. In the definitive pathological exam, macroscopically, it was a brown nodular mass, with a 3.5 cm smooth outer surface and elastic consistency. Histological examination revealed a highly vascularized mass with areas of spindle cell proliferation with perivascular distribution. No cellular atypia, pleomorphism, or necrosis was present. Immunohistochemical analysis was positive for SMA in the perivascular cells and CD34-positive in the blood vessel endothelial cells (Figure 4).

These findings were compatible with an MPC. After 30 months of follow-up, the patient is currently asymptomatic and without signs of recurrence.

## Discussion

We present, to our knowledge, the first case report of an MPC arising from the broad ligament, which was an incidental finding after a routine gynecological evaluation. MPC is a rare entity that is usually benign and consists of a soft tissue tumor that originates from perivascular myoid cells and has a hemangiopericytoma-like vascular pattern [1,6]. Despite being rare, MPCs have been documented in visceral organs, with the first description of an MPC affecting the female genital tract done by Borella et al. in 2019, reporting three cases, two uterine and one arising from the ovary [4,5]. A comprehensive search in PubMed, Google Scholar, and other databases did not reveal any previously documented cases of gynecological MPCs, particularly in the broad ligament. Therefore, we believe this to be the first reported case. The patient was asymptomatic, as these lesions typically present without pain and follow an indolent course. Additionally, they are more commonly observed in middle-aged women [1,2,7].

Diagnosis is often only possible after histological examination, highlighting the diagnostic challenge of this tumor. In our case, ultrasound and MRI revealed a hypervascularized mass within the broad ligament, but these findings were non-specific and raised the possibility of a suspicious lesion. As seen in other case reports, imaging studies such as ultrasound and MRI may reveal a hypervascularized mass, but they often fail to accurately differentiate MPC from other soft-tissue lesions or even malignant tumors. The differential diagnosis of an adnexal mass should always consider the possibility of an ovarian or fallopian tube tumor, with ultrasound and tumor markers playing a crucial role in this assessment. When considering other potential options, in addition to MPC, other diagnoses include mesenchymal tumors such as leiomyomas and solitary fibrous tumors, as well as malignant lesions like sarcomas. Histopathological and immunohistochemical evaluation is essential for achieving an accurate diagnosis [1,2,5]. Imaging findings are, therefore, valuable for localizing the lesion, assessing its size, and planning surgical intervention, but they are insufficient for a definitive diagnosis of this entity. Ultimately, histopathology and immunohistochemistry remain the gold standards for diagnosing MPC. In cases where the imaging characteristics suggest a hypervascular tumor, such as in our patient, particularly with negative neoplastic markers, a middle-aged patient, and no other features of malignancy, MPC should be considered in the differential diagnosis, but definitive diagnosis requires tissue analysis. Histopathologically, MPC exhibits a distinctive concentric perivascular growth pattern of spindle cells. In this case, the tumor showed no signs of nuclear atypia, necrosis, or pleomorphism, which are crucial in distinguishing it from malignant soft tissue neoplasms such as sarcomas. Immunohistochemistry was vital in establishing the diagnosis, as the tumor cells were positive for SMA and negative for desmin, CD34, S100, and keratin, in line with the typical MPC immunophenotype. These immunohistochemical markers aid in excluding other differential diagnoses, including hemangiomas and solitary fibrous tumors [4,7,8].

The primary treatment for MPC is surgical removal. In most cases, complete excision of the tumor is curative with an excellent prognosis. Literature states that recurrence of MPC is rare but can happen; thus, long-term follow-up after tumor removal is recommended [1,4]. Recurrence was documented in two of the three cases of gynecological MPCs, 15 and 17 months after the surgery, suggesting that it may happen when there is an incomplete surgical resection [4]. In another study, 23 out of 46 cases had marginal or incomplete excision, but only two tumors returned locally after one and four years [8]. In our case, the last follow-up, 30 months after the surgery, did not show any signs of recurrence. The patient will be kept under surveillance regarding the fact that our histological report did not provide information concerning the margin’s evaluation and the fact that recurrence can happen years later. Although MPC is usually a benign lesion, malignant MPC has rarely been described, characterized by an infiltrative growth, prominent cytologic atypia, and increased proliferative activity, features that were not present in this case [7-9].

## Conclusions

We describe a rare benign tumor of the female genital tract, MPCs, which, despite being uncommon and generally benign, pose diagnostic challenges due to their hypervascular nature and potential to mimic other soft tissue tumors. We aim to raise awareness for this rare soft-tissue tumor, as it may arise in unexpected locations and be incidentally detected. Given that imaging alone may not be sufficient for a definitive diagnosis, histopathological evaluation and immunohistochemistry are essential for accurate identification. Additionally, we highlight the importance of ongoing surveillance due to the documented potential for recurrence in similar tumors.
